# Association Between Web-Based Patient Portal Messaging and Disease Activity in Inflammatory Bowel Disease: Retrospective Pilot Cohort Study

**DOI:** 10.2196/87518

**Published:** 2026-06-24

**Authors:** Pavan Vemulakonda, Shriansh Singh, Liza Khutsishvili, Anushka Deogaonkar, Valerie S Stark, Marie L Borum

**Affiliations:** 1 Department of Internal Medicine and Primary Care, George Washington University Hospital, 2150 Pennsylvania Avenue NW, Washington, DC, 20037, United States, 1 2027413000; 2 School of Medicine and Health Sciences, George Washington University, Washington, DC, United States; 3 Department of Gastroenterology and Liver Diseases, George Washington University Hospital, Washington, DC, United States

**Keywords:** patient portals, digital health, electronic messaging, electronic health records, telehealth

## Abstract

**Background:**

Inflammatory bowel disease (IBD) requires ongoing monitoring and effective communication between patients and health care providers. Web-based patient portals may enhance engagement, but their association with disease activity remains unclear.

**Objective:**

We aimed to evaluate the association between patient portal messaging frequency and disease activity among patients with IBD.

**Methods:**

We conducted a retrospective cohort study using electronic health record data from an urban academic center. Patients with Crohn disease, ulcerative colitis, or indeterminate colitis were included. Portal messaging frequency over 2 years (July 2023-July 2025) was analyzed. Associations with demographic variables and disease activity were assessed using *χ*^2^ tests (*P*<.05 was considered significant).

**Results:**

Among 442 patients, 347 (78.5%) sent at least one portal message. Patients sent a mean of 5.27 (SD 7.15) messages over 2 years. High messaging frequency was associated with active disease (*P*=.047) and age group (*P*<.001). Patients with active disease were more likely to send ≥16 messages (n=14, 12.5% vs n=18, 6%). No significant associations were found with sex or race.

**Conclusions:**

Higher portal messaging frequency was associated with active disease in IBD. Patient portals may serve as a tool for disease monitoring and early intervention.

## Introduction

Inflammatory bowel disease (IBD), encompassing Crohn disease and ulcerative colitis, is a chronic, relapsing inflammatory disorder of the gastrointestinal tract requiring lifelong management. The global burden of IBD has increased substantially, with rising incidence in both Western and newly industrialized countries, reflecting environmental and lifestyle changes [1]. Achieving sustained remission remains challenging due to unpredictable disease flares and complications.

Contemporary IBD management emphasizes a treat-to-target approach, focusing on tight disease control and early detection of inflammation using clinical, endoscopic, and biomarker-based parameters [2]. However, implementation of this strategy is often limited by delays in identifying symptom exacerbations between clinic visits.

Effective disease control depends not only on pharmacologic therapy but also on timely communication between patients and health care providers. Breakdowns in communication during transitions or between visits have been associated with adverse outcomes, including increased hospitalizations and medical errors [3]. In IBD, early patient-reported symptoms frequently precede objective disease worsening, underscoring the need for accessible communication systems.

Digital health technologies, particularly electronic health record (EHR)–integrated patient portals, have emerged as important tools to facilitate patient engagement and intervisit communication [4]. These platforms allow secure messaging, access to laboratory results, and remote monitoring. Their use expanded significantly during and after the COVID-19 pandemic, accelerating adoption of telemedicine and asynchronous care models [5].

In other chronic diseases, such as diabetes and hypertension, patient portal use has been associated with improved adherence, better disease control, and enhanced patient satisfaction [6-8]. Systematic reviews suggest that patient portals can improve health care processes and some clinical outcomes, although effects vary across settings [6].

However, evidence regarding portal use in IBD remains limited and inconsistent. Studies examining digital health interventions in IBD populations are heterogeneous, with variation in study design, definitions of engagement, and outcome measures. A study by Reich et al [9] demonstrated an association between portal messaging and improved quality of life in IBD patients but did not assess disease activity directly.

Importantly, the relationship between portal messaging behavior and objective disease activity remains unclear. Increased messaging may reflect worsening disease, proactive patient engagement, or both. Additionally, demographic patterns of portal use, including age, sex, and race, have not been consistently characterized in IBD populations.

There is limited evidence evaluating whether patient-initiated portal messaging correlates with disease activity in IBD and whether it can serve as a surrogate marker for clinical worsening. This study aims to evaluate the association between web-based patient portal messaging frequency and disease activity among individuals with IBD while also characterizing demographic patterns of portal use.

## Methods

### Study Overview

A retrospective cohort study was conducted using EHR data from an academic medical center in Washington, DC. Patients with a confirmed diagnosis of Crohn disease, ulcerative colitis, or indeterminate colitis who had at least one outpatient gastroenterology visit during the study period were included. Patients without follow-up within the preceding 3 years were excluded.

### Data Collection and Variables

Demographic variables including age, sex, and race were extracted from the EHR. Clinical data were obtained from physician documentation, endoscopy reports, and patient-reported symptoms recorded in clinic notes.

### Definition of Disease Activity

Disease activity was categorized as active or in remission based on a composite assessment of clinical documentation. Patients were classified as having active disease if the last clinical note indicated ongoing symptoms, disease flare, or escalation of therapy. Patients without such documentation were classified as being in remission or having unknown disease activity.

Because standardized disease activity indices were not consistently available in the retrospective chart review, the study used our definitions of clinical remission and active disease based on physician documentation and treatment escalation. We recognize that this definition is not a universally accepted measure of remission and may introduce variability in classification.

### Portal Messaging Measurement

The EHR includes a web-based patient portal through which patients can send messages to their health care providers. The number of patient-initiated messages sent to gastroenterology care providers over a 2-year period (July 2023 to July 2025) was recorded. Messaging frequency was categorized into predefined groups (0, 1‐5, 6‐10, 11‐15, 16‐20, and >21 messages) based on the distribution of messaging frequency within the cohort.

### Statistical Analysis

Descriptive statistics were used to summarize patient characteristics and messaging patterns. Associations between portal messaging frequency and demographic variables (age, sex, and race) as well as clinical variables (disease activity and IBD subtype) were analyzed using *χ*^2^ tests of association. Statistical significance was defined as *P*<.05.

### Ethical Considerations

This study was approved and deemed exempt by the institutional review board at George Washington University. Informed consent was waived due to the study carrying no more than minimal risk to participants. All data were deidentified and kept in password-protected spreadsheets. Study participants did not receive any compensation.

## Results

### Clinical Characteristics

A total of 442 patients with IBD were included in the analysis. Of these, 182 (41.2%) had ulcerative colitis, 249 (56.3%) had Crohn disease, and 11 (3%) had indeterminate colitis. Regarding race, 196 (44.3%) patients were White, 161 (36.4%) were Black, 7 (2%) were Asian, 28 (6%) were of other races, and 50 (11%) were of unknown race. A total of 253 (57.2%) patients were female and 189 (42.8%) were male. Patients had an in-person or telemedicine visit with a gastroenterology care provider on average 3.2 times during the 2-year study period.

### Portal Messaging Patterns

Overall, 347 of 442 patients (78.5%) sent at least one portal message during the 2-year study period. Patients sent an average of 5.27 messages over this time frame. A total of 201 (45.5%) patients sent 1 to 5 messages, 87 (20%) sent 6 to 10 messages, and 59 (13%) sent 11 or more messages. The distribution of messaging frequency is presented in Table 1.

**Table 1.** Number of patient-initiated portal messages in patients with inflammatory bowel disease (N=442).

Number of patient-initiated portal messages within the previous 2 years	Patients, n
0	95
1 to 5	201
6 to 10	87
11 to 15	27
16 to 20	14
More than 21	18

### Association With Age

Portal messaging frequency varied significantly across age age groups (*P*<.001). Only 16 of 136 (11.8%) patients aged 40 to 60 years sent ≥6 messages over the previous 2 years, in comparison to 64 of 190 (33.7%) patients aged <40 years and 39 of 116 (33.6%) patients aged >60 years (*P*<.001; *χ*^2^_1_=20.6 and *P*<.001; *χ*^2^_1_=17.5). The distribution of messaging frequency by age is presented in Table 2.

**Table 2.** Number of patient-initiated portal messages within the previous 2 years among patients with inflammatory bowel disease by age (N=442).

	Patients by age group (years), n					
Messages, n	<20	20-29	30-39	40-49	50-59	60-69
0	1	12	22	18	10	32
1 to 5	2	39	50	56	36	45
6 to 10	1	13	26	10	2	23
11 to 15	0	5	6	2	0	6
16 to 20	0	4	5	0	1	2
More than 21	0	4	0	1	0	8

### Association With Disease Activity

Messaging intensity also differed by disease activity (*P*=.047; *χ*^2^_1_=3.9), with 14 of 126 (11.1%) patients with active disease sending 16 or more portal messages in the last 2 years in comparison to just 18 of 316 (6%) patients in remission or with unknown disease activity. The distribution of messaging frequency by IBD disease activity is presented in Table 3.

**Table 3.** Number of patient-initiated portal messages within the previous 2 years among patients with inflammatory bowel disease by disease activity (N=442).

Messages, n	Patients in remission, n	Patients with active disease, n	Patients with unknown disease activity, n
0	57	24	14
1 to 5	134	55	12
6 to 10	58	24	5
11 to 15	18	9	0
16 to 20	6	7	1
More than 21	11	7	0

### Association With Sex and Race

No significant associations were observed between messaging intensity and sex, race, or IBD subtype.

## Discussion

### Principal Findings

This study demonstrates that higher patient portal messaging intensity is associated with active disease in individuals with IBD. Additionally, a bimodal distribution of portal engagement was observed, with higher messaging frequency among younger and older patients compared to middle-aged patients. These findings suggest that patient-initiated portal messaging may serve as a behavioral marker of disease activity and engagement with care.

### Comparison With Prior Work

These findings are consistent with prior studies demonstrating increasing use of digital health tools for chronic disease management and the growing role of patient portals in facilitating communication and engagement [4,6]. Previous work has shown associations between portal use and improved patient-reported outcomes, including quality of life in IBD populations [9]. However, most prior studies have not directly examined disease activity. This study extends the existing literature by demonstrating a measurable association between portal messaging behavior and clinical disease status.

The observed bimodal age distribution aligns with findings from prior studies in both IBD and other chronic disease populations. Increased engagement among younger individuals may reflect greater familiarity with digital technologies, whereas higher engagement among older patients may be driven by increased health care needs and comorbidity burden [6,7].

An important observation in this study is the lower level of engagement among middle-aged patients (aged 40‐60 years), which contrasts with the higher engagement seen in both younger and older groups. This finding may reflect competing professional and personal responsibilities that limit engagement with health care systems in this population. Alternatively, it may indicate differences in digital health adoption or perceived need for care. While not the primary focus of the study, this pattern highlights a potential gap in digital engagement that warrants further investigation.

Additionally, although higher messaging intensity was associated with active disease, the directionality of this relationship cannot be established. Increased messaging may reflect reactive communication in response to worsening symptoms, proactive engagement in disease management, or a combination of both.

It is also possible that a subset of portal messages reflected symptoms attributable to overlapping irritable bowel syndrome (IBS) rather than IBD activity alone. Because message content was not systematically reviewed, differentiation between IBS-related and IBD-related symptoms was not possible with the current study design. This represents an important limitation and an area for future investigation.

### Clinical Implications

These findings suggest that portal messaging may serve as a low-cost and scalable indicator of disease activity in IBD. Increased messaging frequency could potentially be used to identify patients at higher risk of disease exacerbation and to prompt earlier clinical intervention.

From a patient-centered perspective, high message volume may also indicate unmet clinical needs or inefficiencies in communication workflows. For example, some patients with active disease required more than 16 messages over a 2-year period, which may suggest opportunities for improved triage systems, nurse-led protocols, remote symptom monitoring tools, or earlier clinical follow-up to address concerns more efficiently.

Furthermore, the identification of lower engagement among middle-aged patients highlights an opportunity for targeted interventions aimed at improving digital participation and ensuring equitable access to care across age groups [4,6].

Portal use may also reflect patient preference for asynchronous communication compared with telephone-based communication. Factors contributing to portal use may include convenience, faster documentation of concerns, accessibility outside clinic hours, and integration of laboratory and medication records into the EHR.

### Limitations

This study has several limitations. First, as a single-center retrospective study, the findings may not be generalizable to other health care settings with different patient populations or care delivery models. Second, disease activity was determined based on clinical documentation rather than standardized indices, which may introduce variability in classification. Third, the content of portal messages was not analyzed, limiting the ability to distinguish between clinically relevant and administrative communication. Fourth, patients who did not activate the web-based patient portal were included in the study, likely impacting the distribution of portal messages. Finally, the temporal relationship between messaging and disease flares was not assessed, precluding conclusions about causality.

Additionally, a baseline comparison of portal use rates among non-IBD gastroenterology populations was not available for this study, limiting contextual interpretation of whether the observed messaging frequencies were unique to patients with IBD or reflected broader portal use trends in gastroenterology practice.

Future research should focus on multicenter studies to validate these findings across diverse populations. Prospective studies examining the temporal relationship between portal messaging and disease activity could help clarify causality. Additionally, qualitative analysis of message content may provide insight into the types of communication that are most predictive of clinical deterioration. Interventional studies aimed at enhancing portal engagement, particularly among underrepresented groups such as middle-aged patients, may further improve patient outcomes.

### Conclusions

In conclusion, patient portal messaging frequency is associated with disease activity in individuals with IBD. This finding suggests that digital communication patterns may serve as an important adjunct for disease monitoring and patient-centered care. Leveraging patient-portal data may enhance early detection of disease exacerbations and improve overall clinical management in IBD.

Given the exploratory nature of this work and the study’s retrospective design, these findings should be interpreted as hypothesis-generating and supportive of future prospective validation studies.

## Abbreviations

EHR: electronic health record
IBD: inflammatory bowel disease
IBS: irritable bowel syndrome
